# Spermine Enhances the Peroxidase Activities of Multimeric Antiparallel G-quadruplex DNAzymes

**DOI:** 10.3390/bios15010012

**Published:** 2025-01-02

**Authors:** Raphael I. Adeoye, Theresia K. Ralebitso-Senior, Amanda Boddis, Amanda J. Reid, Francesca Giuntini, Amos A. Fatokun, Andrew K. Powell, Adaoha Ihekwaba-Ndibe, Sylvia O. Malomo, Femi J. Olorunniji

**Affiliations:** 1 School of Pharmacy & Biomolecular Sciences, Faculty of Health, Innovation, Technology and Science, Liverpool John Moores University, Liverpool L3 3AF, UK; r.i.adeoye@ljmu.ac.uk (R.I.A.);; 2 Department of Biochemistry, University of Ilorin, Ilorin 240003, Kwara State, Nigeria; 3 School of Life Sciences, Coventry University, Coventry CV1 2DS, UK

**Keywords:** G-quadruplex, DNAzymes, spermine, multivalent G-quartets, biosensing, nanozymes

## Abstract

G-quadruplex (G4) DNAzymes with peroxidase activities hold potential for applications in biosensing. While these nanozymes are easy to assemble, they are not as efficient as natural peroxidase enzymes. Several approaches are being used to better understand the structural basis of their reaction mechanisms, with a view to designing constructs with improved catalytic activities. Spermine alters the structures and enhances the activities of some G4 DNAzymes. The reported effect of spermine in shifting the conformation of some G4 DNAzymes from antiparallel to parallel has not been tested on multimeric G4 DNAzymes. In this study, we examined the effects of spermine on the catalytic activities of multivalent constructs of Bcl2, c-MYC, PS2.M, and PS5.M. Our findings show that spermine significantly improved the peroxidase activity of PS2.M, an antiparallel G4 DNAzyme, while there was no significant effect on c-MYC, which already exists in a parallel conformation. The addition of spermine led to a substantial increase in the initial velocity of PS2.M and its multimeric form, enhancing it by approximately twofold. Therefore, spermine enhancement offers promise in expanding the range of DNAzymes available for use as biosensing tools.

## 1. Introduction

DNAzymes, also known as deoxyribozymes, are catalytic DNA structures that can mediate reactions that are usually associated with catalysis by protein enzymes [1]. One group of such DNA nanostructures are guanine-rich oligonucleotides capable of complexing with hemin (the cofactor of many oxidase enzymes) to form G-quadruplexes (G4) with peroxidase mimetic activity [2,3,4].

**Figure 1:**
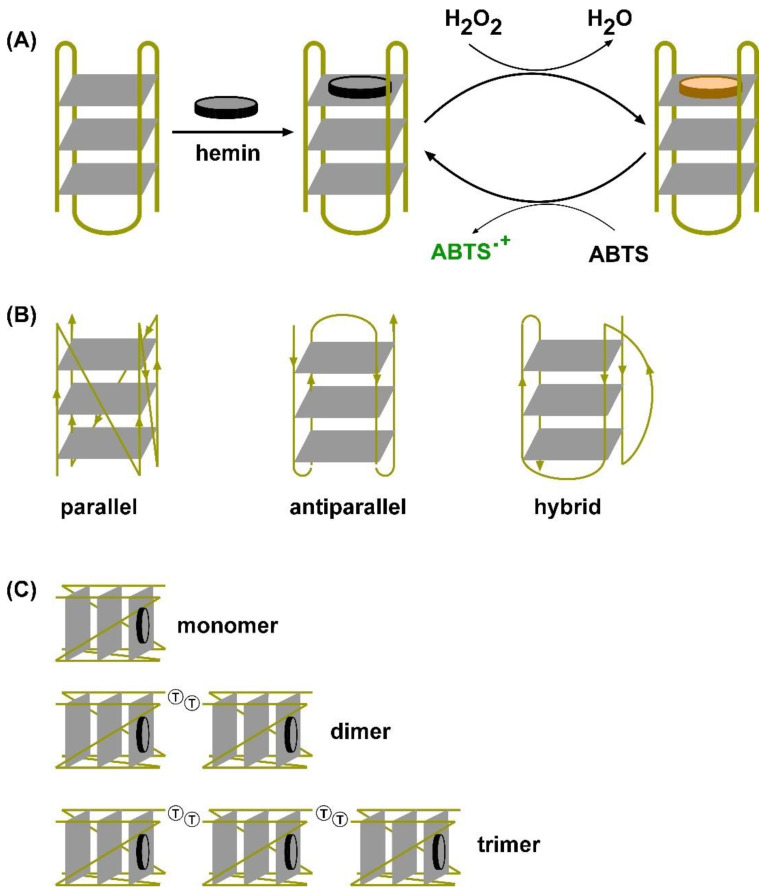
Structure and mechanism of G-quadruplex DNA reactions. (**A**) Assembly of G-quadruplex-hemin DNAzymes and H_2_O_2_-activated oxidation of ABTS: Guanine-rich DNA oligonucleotides fold to form G-quartet interactions (grey planes) which bind to hemin (grey discs). The resulting DNAzyme complex activates H_2_O_2_ to oxidise a donor substrate (in this instance ABTS) using a mechanism like haem peroxidase-catalysed reactions. The reduced haem (brown disc) can react with H_2_O_2_ and initiate another round of ABTS oxidation. The reaction product (ABTS^•^) has a bright green colour with an absorption maximum around 412–422 nm. (**B**) Topologies of G-quadruplexes: G-qudruplexes fold into parallel, antiparallel, or hybrid complexes depending on the DNA sequence and the buffer composition in which they are formed. (**C**) Schematic illustrations of the design of G-quadruplex DNAzyme dimers and trimers. In the multimers, each monomeric unit is joined to the next by a TT dinucleotide.

Guanine-rich oligonucleotides can fold into G-quartet structures through Hoogsteen hydrogen bonds in the presence of metal ions [5]. Several G-quartets can assemble to form four-stranded DNAs known as G-quadruplexes (Figure 1). When these G4 structures are complexed with hemin, they display peroxidase activity by using hydrogen peroxide as an oxidising substrate [6,7]. G-rich sequences are found abundantly in several important genomic DNA regions such as telomeres and the promoter regions of human genomes, where they easily adopt a quadruplex structure to maintain the integrity of the chromosome [8]. It has been reported that 400,000 G4 sequences are found in the human genome, and some of these promoters have been identified to control the expression of proto-oncogenes, like the promoter of c-MYC and Bcl-2 [9].

Peroxidase-mimicking DNAzymes have found applications in the development of biosensors. However, their low catalytic activity and the instability of the reaction product of the chromogenic substrate limit the potential applications of these innovative systems [10]. Several studies have been conducted to enhance the catalytic activity of peroxidase-mimicking DNAzymes. These include the addition of additives such as ATP [11,12] and biogenic polyamines such as spermine and spermidine [13]. Other approaches involve the use of specific flanking sequences such as d(AAA) and d(CCC) in the G4 [4,14,15], multimerisation [7,10,16], covalent conjugation of cationic peptide [17], and functionalized modification of hemin [18]. Despite these efforts, the catalytic activities of DNAzymes still remain relatively low.

G-quadruplexes can take on different topologies such as antiparallel, parallel, or mixed parallel (Figure 1C). The specific topology formed depends on the type of metal ion and the DNA oligonucleotide sequence [19]. The activity of the G-quadruplex formed is determined by its topology [13]. Antiparallel G-quadruplexes, both inter and intramolecular, exhibit low peroxidase activity. Intermolecular parallel conformation also results in low peroxidase activity, while parallel and mixed parallel intramolecular quadruplexes exhibit high peroxidase activity [19].

Biological polyamines such as spermine, spermidine, and putrescine are widely found in organisms and tissues, and they play a role in regulating gene expression, facilitating DNA–protein interactions and protecting DNA molecules from damaging agents [20].

When polyamines are combined with G-quadruplexes (G4s), they alter the structure of G4s and increase their peroxidase activity [13]. Previous studies have shown that polyamines can cause the transition of monomeric G4 DNAzymes c-MYC and c-Kit from an antiparallel to a parallel conformation [21,22]. Multimeric DNAzymes are enzymes containing several identical units linked together to form multivalent units with multiple active sites (Figure 1C). It has been reported that multimeric G4s exhibit improved activity compared to the monomeric form due to the synergistic effect of the individual units and the creation of an additional high-affinity hemin bind [10,23].

While different strategies have been developed to optimise the activities of G-quadruplex peroxidases, few studies have examined how these different approaches could affect the performance of these systems when they are combined in the design and/or the operation of the DNAzymes. Previous studies have shown that multimerization alone has limited impact on the rate enhancement of DNAzymes. Considering this limitation and the need to explore further applications of these enzymes, this study investigated the ability of spermine and ATP to enhance the intrinsic peroxidase activity of the monomers, dimers, and trimers of the four G-quadruplex DNAzymes.

Multimeric enzymes consist of multiple monomers of the same unit and have several active sites, which give them greater activity compared to the monomeric form. However, for multimers to offer enhanced catalytic activity, they must be in the correct topology [24]. In previous studies carried out by our group and others [10,23,25], multivalent DNAzymes (dimers and trimers) were constructed by using a dinucleotide linker, TT, to connect one end of the oligonucleotide to another.

In this study, we examined how spermine affects the catalytic activities of various G-quadruplexes, including Bcl2, c-MYC, PS2.M, and PS5.M. We focussed on how multimerisation of the G4 units influence the rate enhancements reported for spermine and ATP. Specifically, we want to understand how the addition of spermine impacts the activities of these G-quadruplexes in relation to their parallel or antiparallel structures, as well as to determine if there are differences in how multimers respond to the additives. Understanding these effects could help enhance the catalytic activities of G-quadruplex enzymes and expand their applications in biosensor development.

## 2. Materials and Methods

### 2.1. Reagents and DNA Oligonucleotides

HPLC purified oligonucleotides were obtained from Eurofins MWG Operon, Ebersberg, Germany, and were used without further purification. All other reagents used were obtained from Fisher Scientific, Loughborough, UK, and were of analytical grade unless otherwise specified. Oligonucleotides were dissolved in TE buffer (10 mM Tris-HCl pH 7.5, 0.1 mM EDTA) as 100 µM solutions and stored at −20 °C until when needed for reactions.

### 2.2. Preparation of DNAzyme (G4–Hemin Complex)

Assembly of the G4–Hemin complex was carried out as reported previously by Adeoye et al. [10]. Briefly, oligonucleotides (Table 1) were complexed to hemin to obtain a catalytic unit (DNAzyme) with peroxidase activity. Also briefly, 50 µL of 100 µM oligonucleotide solution in TE (1×) was added to 450 µL of a reaction buffer (RB) containing 25 mM HEPES-NaOH pH 7.4, 20 mM KCl, 200 mM NaCl, 0.05% Triton X-100 and 1% DMSO. The oligonucleotide samples were heated at 95 °C for 10 min and cooled rapidly at 0 °C for 15 min. They were later kept at 25 °C for 15 min to attain a higher-ordered structure needed to bind hemin to form a DNA–hemin complex. Thereafter, a 20 μL sample of 100 μM hemin solution was added to 230 μL of 10 μM oligonucleotide, and the resultant solution was mixed properly and was kept at 25 °C for 30 min to allow the formation of the oligonucleotide–hemin complex. The resultant solution gives 10 μM DNAzymes.

### 2.3. Measurement of Peroxidase Activity

DNAzyme reacts with H_2_O_2_ to generate an activated complex that oxidises a chromogenic organic substrate. The activities of the G4/hemin DNAzymes were determined as reported previously [10]. Reactions were carried out at 25 °C in reaction buffer containing 25 mM HEPES-NaOH pH 7.4, 20 mM KCl, 200 mM NaCl, 0.05% Triton X-100 and 1% DMSO. The G4–Hemin DNAzyme (0.25 µM) and the organic chromogenic substrate ABTS (2.5 mM) were mixed and incubated at 25 °C before initiation of the reaction with the addition of 425 µM H_2_O_2._ The reaction progress was monitored for 5 min on a CLARIOstar Plate Reader (BMG LABTECH), and readings were taken every 5 s by measuring the increase in absorbance at λ = 415 nm (ΔA_415_) of the reaction product (ABTS•).

**Figure 2:**
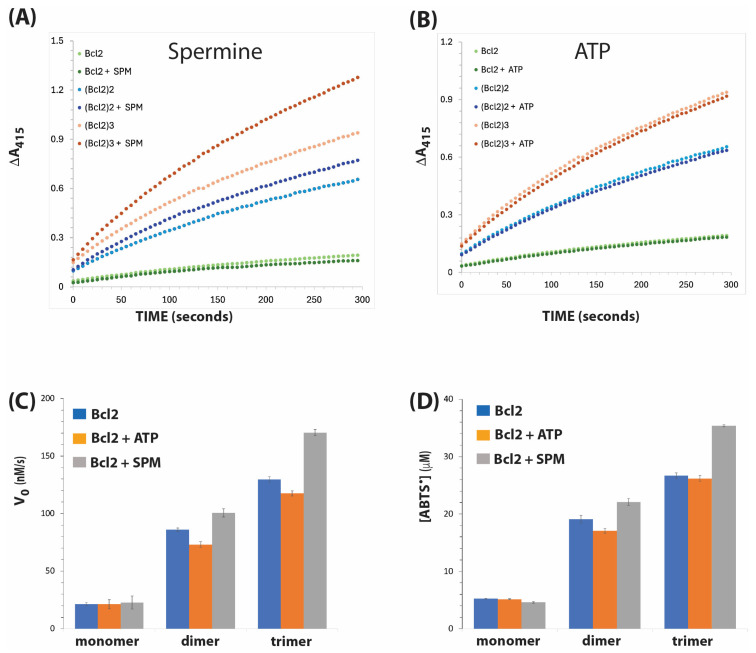
DNAzyme activities of Bcl-2 in the presence of spermine and ATP. Time courses of reactions are shown for spermine (1 mM) in (**A**) and for ATP (1 mM) in (**B**). The absorbance values were adjusted using blank reactions in which DNAzyme was not included. (**C**) Initial velocity (expressed as Vo, nM/s) was calculated from the slope of the linear portion (usually, the first 30 s) of the time course. (**D**) The amount of ABTS radical produced after 300 s from the activity of 0.25 µM G4/DNAzyme of (**A**). In (**C**,**D**), each value represents the mean ± standard deviation of four measurements.

**Figure 3:**
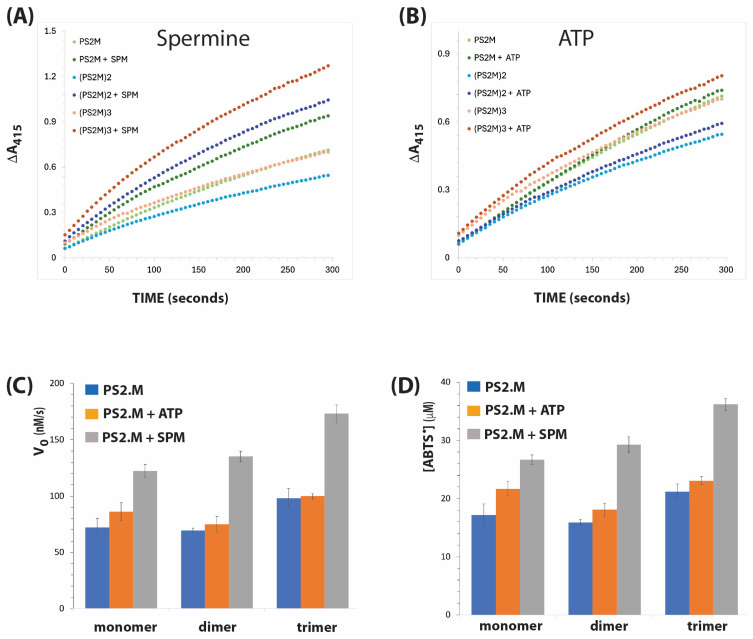
DNAzyme activities of PS2.M in the presence of spermine and ATP. Time courses of reactions are shown for spermine (1 mM) in (**A**) and for ATP (1 mM) in (**B**). (**C**) Initial velocity (expressed as Vo, nM/s). (**D**) The amount of ABTS radical produced after 300 s. Reaction conditions and analyses are as described in Figure 2.

**Figure 4:**
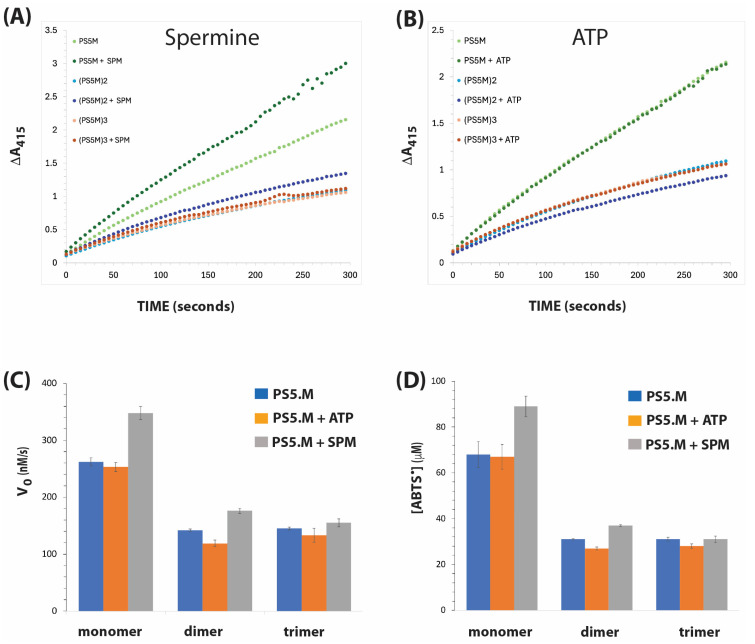
DNAzyme activities of PS5.M in the presence of spermine and ATP. Time courses of reactions are shown for spermine (1 mM) in (**A**) and for ATP (1 mM) in (**B**). (**C**) Initial velocity (expressed as Vo, nM/s). (**D**) The amount of ABTS radical produced after 300 s. Reaction conditions and analyses are as described in Figure 2.

**Figure 5:**
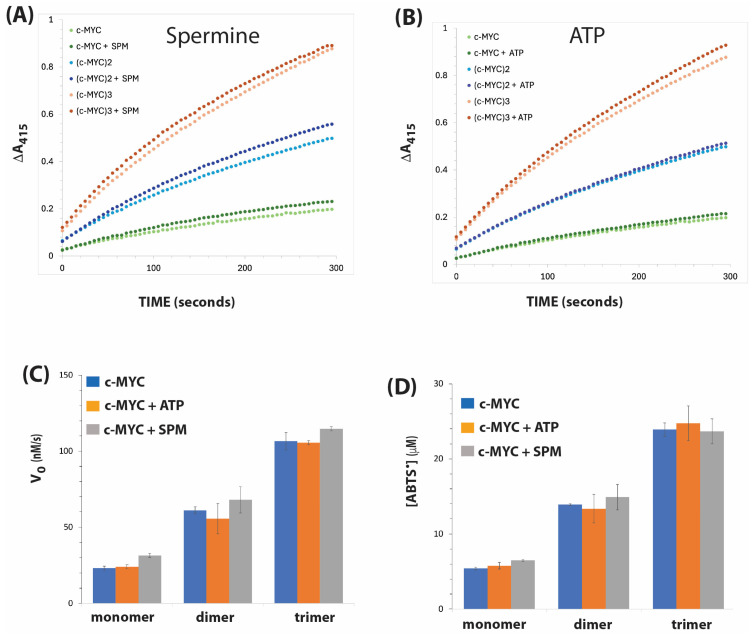
DNAzyme activities of c-MYC in the presence of spermine and ATP. Time courses of reactions are shown for spermine (1 mM) in (**A**) and for ATP (1 mM) in (**B**). (**C**) Initial velocity (expressed as Vo, nM/s). (**D**) The amount of ABTS radical produced after 300 s. Reaction conditions and analyses are as described in Figure 2.

To test the effect of exogenous enhancers, aqueous solutions of spermine or ATP were added to a final concentration of 1 mM spermine or 1 mM ATP, before the initiation of the reaction with the addition of 425 µM H_2_O_2_. We carried out preliminary experiments to determine the optimal concentrations of spermine using monomeric PS2.M. The results show that the best activities were obtained at a final concentration of 1 mM. To ensure valid comparisons of activities at equimolar concentrations, we used the same concentrations for spermine and ATP in the experiments shown in Figure 2, Figure 3, Figure 4 and Figure 5.

A molar extinction coefficient of 36,000 M^−1^cm^−1^ for ABTS• was used to convert absorbance changes to the amount of ABTS• formed (10). The initial reaction rate, Vo, expressed as nM/s, and the amount of ABTS• formed after 5 min of reaction, expressed as nM/s, were determined from the time course of the reaction. The Vo was calculated using linear regression functions in Microsoft Excel from the slope of the initial (30 s) linear portion of the ΔA_415_ [17,19].

## 3. Results

### 3.1. Experimental Approach

To assess the ability of ATP and spermine (a polycationic amine) to enhance the activities of G-quadruplex DNAzymes, we chose four well-characterised G-quadruplex DNAzymes. These are Bcl2, PS2.M, PS5.M, and c-MYC (Table 1). The DNAzymes, PS2.M and PS5.M, were obtained in the laboratory through an in vitro reiteration process [6]. PS2.M and PS5.M are among the most extensively studied G4 DNAzymes.

The genes c-MYC and Bcl-2 are naturally found in G-rich segments of chromosomes. c-MYC is a group of proto-oncogenes and regulator genes that encode transcription factors, whereas Bcl-2 is overexpressed in cancer cells and inhibits pro-apoptotic signals to enable cancer cells to survive in adverse conditions [26,27]. The short G4-forming sequences derived from these genes have been shown to have peroxidase activities.

The catalytic activity of peroxidase-mimicking G-quadruplex DNAzymes is limited, and this has restricted their use in biosensor development. Studies have shown that parallel G4s provide better hemin-binding sites compared to antiparallel G4s [24,28,29]. In antiparallel G4s, loops partially block hemin cofactor interactions with the G-quartet and may also shield the hemin, making it less accessible to substrates [14,28]. Optimisation of the unique structure and resilience of parallel G4s is one way through which the catalytic activities of G-quadruplex DNAzymes could be improved. We hypothesised that spermine or ATP binding to G4 structures could change the conformation of these DNAzymes from antiparallel structures to more active parallel structures, potentially enhancing their peroxidase activity. Additionally, we extended our previous work on DNAzyme multimers [10] to investigate whether the additives known to enhance peroxidase activities interact the same way with monomers and multimers.

### 3.2. Catalytic Activities of Bcl-2 Constructs

The activities of Bcl-2 constructs and the effects of ATP and spermine are shown in Figure 2. Consistent with our previous report, the peroxidase activity of Bcl-2 was enhanced by multimerisation [10]. The initial velocities for the Bcl-2 monomer, dimer, and trimer were 21.3, 86.1, and 129.6 nM/s, respectively. This represents a rate enhancement by four- and sixfold for the Bcl-2 dimer and trimer, respectively. Spermine significantly increased the initial velocity of Bcl-2 trimer from 129.6 nM/s to 170.4 nM/s; however, there was no significant improvement of Bcl-2 monomer by spermine (Figure 2C).

Similarly, spermine enhanced the total amount of ABTS radical formed after 300 s of reaction (Figure 2A) with the Bcl-2 dimer and trimer by approximately five- and eightfold, respectively, when compared to the control multimers in which spermine was not added (Figure 2D). However, unlike spermine, ATP did not enhance the activity of Bcl-2 monomers and multimers.

### 3.3. Catalytic Activities of PS2.M Construct

The catalytic activities of PS2.M monomer, dimer, and trimer constructs, and the effects of the additives ATP and spermine are shown in Figure 3. The activities of the monomer and dimer are similar, while the trimer showed enhanced activity compared to the monomer. The initial velocity of PS2.M monomer was 72.2 nM/s, while its activities in the presence of ATP and spermine were 86.1 nM/s and 122.2 nM/s, respectively. Though multimerisation did not significantly change the catalytic activity of PS2.M, the addition of spermine offers remarkable improvement in the activity of PS2.M and its multimeric form by approximately twofold enhancement. The reaction extents of ABTS oxidation were 17.2, 21.7, and 26.7 µM/s for PS2.M, PS2.M + ATP, and PS2.M + SPM, respectively (Table 2). Similarly, the addition of spermine enhanced the reaction extent of (PS2.M)2 from 15.9 to 29.3 µM, and that of (PS2.M)3 from 21.2 to 36.2 µM, respectively. However, ATP only offered moderate improvements in both the initial velocity and extent of ABTS oxidation by PS2.M monomer, dimer, and trimer (Figure 3).

### 3.4. Catalytic Activities of PS5.M Construct

Figure 4 shows the catalytic activities of the PS5.M monomer, dimer, and trimer constructs, and the effects of the additives ATP and spermine. In contrast to Bcl-2 and PS2.M, multimerisation did not improve the inherent peroxidase activity of PS5.M. Additionally, the presence of ATP did not enhance the initial velocity of the PS5.M monomer, dimer, and trimer. However, the addition of spermine led to an increase in the initial rate of the reaction from 262 to 348 nM/s. Spermine also slightly enhanced the initial speed of PS5.M dimer and trimer. Furthermore, the addition of spermine to PS5.M monomer notably increased the amount of ABTS radicals formed from 68 to 89 µM after 300 s (Figure 4D).

### 3.5. Catalytic Activities of c-MYC Construct

Figure 5 shows the catalytic activities of c-MYC monomer, dimer, and trimer constructs, and the effects of the additives ATP and spermine. The initial velocity of ABTS oxidation of the c-MYC dimer was threefold higher than that of the monomer, and the trimer was increased by almost fivefold. The same pattern is seen in the observed amount of product formed after 5 min of reaction. The addition of ATP did not enhance either the initial rates or the extent of the ABTS oxidation reaction for any of the three c-MYC constructs. The addition of spermine slightly enhanced the initial rate of ABTS oxidation of the c-MYC monomer and trimer, but not for the dimer. However, there was no significant rate enhancement in the extent of the reaction following the addition of spermine to the dimer and trimer of c-MYC.

## 4. Discussion

We have previously shown that multimerisation can enhance the peroxidase activities of G-quadruplex DNAzymes [10]. Other researchers have shown that adding certain agents such as spermine, spermidine, and ATP can enhance the peroxidase activity of monomeric DNAzyme [11,12,13]. These studies indicate that the full range of factors that could potentially enhance the activities of G4 DNAzymes is still not fully understood.

We determined the initial velocity (Vo) and the amount of ABTS peroxidation product ([ABTS•]) formed after 5 min for each of the four DNAzymes (Table 2). This could allow us to understand the mechanism through which spermine or ATP enhanced the activities of the DNAzymes. For example, the reaction of the ABTS• radical cation with itself or with H_2_O_2_ could lead to its disproportionation [30,31,32]. Such a pattern can be seen by analysing the time course of the reaction and by comparing the initial rate with the amount of ABTS peroxidation product remaining at the end of the reaction [10].

In this work, we studied how these small molecule activators interact with both monomeric and multimeric DNAzymes. We found that while PS2.M displays low peroxidase activity, the activities of both monomeric and multimeric PS2.M were enhanced by the addition of spermine (Table 2). This enhancement may be due to a change in the DNAzyme’s conformation. Furthermore, the G-quadruplex loops in the parallel and mixed parallel topologies with better stacked hemins allow better access for hemin to the oxidising substrate or organic reductant, unlike in the antiparallel topology where the hemin is covered and less accessible to the substrate [19,28,29].

Qi et al. showed that spermine provided a favourable environment for the DNAzyme by condensing the G4 structures and by protecting the hemin cofactor from rapid inactivation, resulting in an increase in its peroxidase activity [13]. Additionally, Nakayama and Sintim reported that certain guanine-quadruplex structures have a higher affinity for binding amino group-rich peptides compared to double or single-stranded DNAs [19]. Zhou et al. also reported that ammonium ions enhanced the activity of the G4 DNAzyme [7]. This could explain the effect of spermine (a compound-containing amino group) on the activities of PS2.M, wherein it influences a structural change that enhances the catalytic activity of PS2.M. It is likely that spermine induces changes in the topology or structure of G4, leading to an increased affinity for hemin, and subsequently enhancing catalytic activity. A similar transition from an antiparallel to parallel topology was also observed in c-kit by Wen and Xie [22].

Parallel G4 structures are more effective at binding hemin compared to antiparallel G4 structures [28,29]. The loops in antiparallel G4 structures partially obstruct hemin’s ability to interact with the G-quartet and may also shield the hemin, making it less accessible to substrates [15,29,33]. Spermine may alter hemin’s binding state to PS2.M, potentially enhancing the catalytic activity of the G4/hemin DNAzyme. Zhou et al. showed that addition of NH_4_^+^ to a dimeric G4 system increases the affinity of hemin to the structure [7]. Liu et al. (2020) observed that modifying the hemin cofactor with an amino group led to a ninefold increase in G4 DNAzyme peroxidase activity [18].

Consistent with our previous observation that multimerisation did not enhance, but rather inhibited, the activities of PS5.M, neither spermine nor ATP had any enhancement effects on (PS5.M)2 and (PS2.M)3 [10]. In contrast, the activities of monomeric PS5.M were slightly boosted by spermine. This observation raises questions regarding the structures of multimeric PS5.M constructs and the effects of multimerisation. It is likely that clustering PS5.M units as multimers limits access to the catalytic centre of the G-quadruplex/hemin structure. Experiments designed to provide more mechanistic insights would help address this intriguing observation.

Another possible mechanism is that spermine prevents hemin from undergoing oxidative damage by sequestering it in the quadruplex structure, protecting it from degradation caused by reactive oxygen species produced by hydrogen peroxide, consequently resulting in an increase in their peroxidase activity [19]. This could explain the effect of spermine on Bcl2 (Figure 2) and PS2.M (Figure 3), where the addition of the polycation consistently boosted the activities of the DNAzymes in monomer, dimer, and trimer forms. Protection of hemin over several minutes during the reaction could allow its turnover, regenerating compound I, the intermediate that initiates the reaction with the reductant substrate. Qi et al. [13] reported that the addition of spermine to the PS2.M/hemin complex resulted in a significant broadening and hyperchromicity of the Soret band of the porphyrin in the absorption spectra. They reported that spermine influences tight binding of hemin to the G4 DNAzyme through the provision of a hydrophobic binding site that stabilises ferryl heme intermediates. Additionally, the amino group of spermine could form hydrogen bonds with carbonyl groups of the G-tetrads, interacting with its substrate to form a more stable intermediate and transition state species involved in the formation of compound I in the classical peroxidase cycle [7].

In contrast to the effect on PS2.M, spermine did not have much effect on c-MYC, probably because c-MYC was already in the parallel conformation. Kumar et al. [21] reported that c-MYC predominantly exists in a parallel conformation in the presence of K^+^, which was the cofactor present in our assay mixture.

We also tested the effect of ATP on the four DNAzymes; moderate rate enhancement was only observed in PS2.M monomers. However, we did not observe any positive effect of the nucleotide on the activities of all the other three DNAzymes either in their monomeric or multimeric forms. Kong et al. [11] and Stefan et al. [12] reported ATP boosting activity for CatG4 and 22AG DNAzymes, respectively. The differences observed in ATP boosting activity in our study might be due to differences in the oligonucleotide sequences and steric hinderances in the multimers which either obstruct hemin’s ability to interact with the G-quartet or shield the hemin, consequently making it less accessible to substrates.

They reported that ATP enhanced DNAzyme activity by functioning as an H_2_O_2_ activator, electron transfer facilitator, and a stabilising agent, with all these functions being intricately linked. The nucleoside of ATP accepts a proton, thereby facilitating the binding of H_2_O_2_ to iron (III) protoporphyrin. It acts as a dual stabilising agent by protecting the hemin/hemin–oligonucleotide complex and stabilising the cation radical produced by the chromogenic substrate. ATP’s boosting capability may be because it protects hemin/the hemin–G-quadruplex [11]. They also showed that ATP increases the binding affinity of hemin to G4 DNAzyme by causing a significant shift in the hyperchromicity of the hemin’s Soret band upon its addition to the hemin–G4 DNAzyme complex. Hence, catalysis can continue at a high rate even at high hydrogen peroxide concentrations. It has also been reported that the presence of ATP enhances the temperature stability of the G-quadruplex enzyme by protecting various reaction components [12]. Overall, the pattern of results shows that unlike spermine, ATP did not have a remarkable effect on the activities of the DNAzymes we studied in this work, either in monomeric or multimeric forms.

## 5. Conclusions

In this study, we demonstrated that the addition of spermine to antiparallel multimeric DNAzymes can facilitate their conversion into catalytically active parallel conformations. The significant increase in both the initial reaction velocity and the overall reaction extent, when comparing the multimeric form (PS2.M) to the monomeric form (Table 2), indicates its potential application in biosensors. Furthermore, these findings shed light on the possibility of enhancing the activities of G4 DNAzymes with low catalytic activities into active forms. Highly active G4 DNAzymes with peroxidase-mimicking activity offer opportunities for developing non-invasive, accurate, sensitive, specific, and cheap point-of-care biosensors for early detection of different diseases including various strains of coronavirus. To gain a deeper understanding of the catalytic mechanisms involved in this activation, further structural analysis is required. Additionally, more comprehensive studies on other antiparallel G4 DNAzymes are needed to determine whether the enhancement observed with spermine is a universal phenomenon.

## Figures and Tables

**Table 1:** Sequences of DNAzyme oligonucleotides used in this study.

Oligonucleotides	Sequence
Bcl-2	5′GGGCGCGGGAGGAAGGGGGCGGG3′
PS2.M	5′GTGGGTAGGGCGGGTTGG3′
PS5.M	5′GTGGGTCATTGTGGGTGGGTGTGG3′
c-MYC	5′GAGGGTGGGGAGGGTGGGGAAG3′

**Table 2:** Initial velocity (Vo (nM/s)) and reaction extent of ABTS oxidation ([ABTS*]) of DNAzymes monomer, dimer and trimer in the absence or presence of ATP or spermine.

	Bcl2		(Bcl2)2		(Bcl2)3	
	Vo	[ABTS•]	Vo	[ABTS•]	Vo	[ABTS•]
DNAzyme alone	21.3 ± 1.3	5.24 ± 0.1	86.1 ± 1.3	19.1 ± 0.7	129.6 ± 1.3	26.7 ± 1.3
DNAzyme + ATP	21.3 ± 3.9	5.17 ± 0.1	71.3 ± 2.6	17.1 ± 0.4	117.6 ± 2.3	26.2 ± 0.5
DNAzyme + Spermine	22.7 ± 5.7	4.61 ± 0.1	100.6 ± 3.5	22.1 ± 0.6	170.4 ± 2.6	35.4 ± 0.2

	**PS2.M**		**(PS2.M)2**		**(PS2.M)3**	
	**Vo**	**[ABTS**•**]**	**Vo**	**[ABTS**•**]**	**Vo**	**[ABTS**•**]**
DNAzyme alone	72.3 ± 7.9	17.2 ± 1.9	69.4 ± 2.3	15.9 ± 0.5	98.1 ± 8.6	21.2 ± 1.3
DNAzyme + ATP	86.1 ± 8.2	21.7 ± 1.2	75.0 ± 6.8	18.1 ± 1.1	100.0 ± 2.3	23.1 ± 0.7
DNAzyme + Spermine	122.2 ± 6.0	26.7 ± 0.8	135.1 ± 4.7	29.3 ± 1.3	173.0 ± 8.0	36.2 ± 1.0
						
	**PS5.M**		**(PS5.M)2**		**(PS5.M)3**	
	**Vo**	**[ABTS**•**]**	**Vo**	**[ABTS**•**]**	**Vo**	**[ABTS**•**]**
DNAzyme alone	262 ± 7.3	68.0 ± 5.7	142 ± 2.3	31.0 ± 0.3	145 ± 2.6	31.0 ± 1.3
DNAzyme + ATP	253 ± 8.2	67.0 ± 5.4	119 ± 5.7	27.0 ± 0.6	133 ± 12.0	28.0 ± 0.7
DNAzyme + Spermine	348 ± 11.6	89.0 ± 4.9	176 ± 4.7	37.0 ± 0.4	155 ± 6.9	31.0 ± 1.0

	**c-MYC**		**(c-MYC)2**		**(c-MYC)3**	
	**Vo**	**[ABTS**•**]**	**Vo**	**[ABTS**•**]**	**Vo**	**[ABTS**•**]**
DNAzyme alone	23.1 ± 1.3	5.4 ± 0.2	61.1 ± 2.3	13.9 ± 0.1	106.5 ± 5.7	23.9 ± 0.9
DNAzyme + ATP	24.1 ± 1.3	5.8 ± 0.5	55.6 ± 9.9	13.4 ± 1.9	105.6 ± 1.4	24.8 ± 2.3
DNAzyme + Spermine	31.5 ± 1.3	6.5 ± 0.1	68.0 ± 8.6	14.9 ± 1.7	114.8 ± 1.3	23.7 ± 1.7

## Data Availability

Data are contained within the article.
